# Clinical Profile of Obstetric Patients Admitted to the Medical-Surgical Intensive Care Unit (MSICU) of an Inner-City Hospital in New York

**DOI:** 10.4021/jocmr1079w

**Published:** 2012-09-12

**Authors:** Jose Orsini, Ashvin Butala, Lizmer Diaz, Eliza Muzylo, Carlo Mainardi, Paul Kastell

**Affiliations:** a Department of Medicine, New York University School of Medicine at Woodhull Medical and Mental Health Center, 760 Broadway, Brooklyn, New York, USA; b Department of Obstetrics and Gynecology, New York University School of Medicine at Woodhull Medical and Mental Health Center, 760 Broadway, Brooklyn, New York, USA

**Keywords:** Obstetric patients, Intensive care units, Intermediate obstetric units, Post-anesthesia care units

## Abstract

**Background:**

Pregnancy is associated with physiological and anatomical changes that usually occur uneventfully in majority of women. However, these changes can cause major maternal morbidity with potential catastrophic consequences. The purpose of this study is to evaluate the clinical characteristics of obstetric patients admitted to the MSICU of an inner-city hospital in New York.

**Methods:**

A prospective, observational study was conducted among all the obstetric patients admitted to the MSICU between June 1, 2009 and June 30, 2012.

**Results:**

A total of 19 obstetric patients were admitted to the MSICU between June 1, 2009 and June 30, 2012. The most common comorbidity on admission was hypertensive disorder. Hemodynamic unstability and shock was the most common admission diagnosis. The mean length of stay was 3.5 days. One patient died.

**Conclusions:**

Obstetric hemorrhage and pregnancy-induced hypertensive disorders remains as the most common entities requiring intensive care unit (ICU) admission among obstetric patients. A multidisciplinary team involvement is essential in the management of these patients.

## Introduction

The need for critical care support and admission to ICU’s in obstetrics patients is relatively infrequent, as they are usually a young and healthy group of patients. Despite the advances in the standard of care in the ICU’s in the recent years, the pregnant patient with medical complications represents a challenge to critical care medicine physicians and often requires a multidisciplinary team involvement. Different approaches to the classification of severe maternal morbidity have been used, including clinically defined morbidities [1], organ system dysfunction [2], and management-based criteria such as the need for intensive care [3]. Hemorrhage and hypertensive disorders constitute most of the obstetric ICU admissions [4]. In spite of the low incidence of admission to critical care units among this population, the overall maternal mortality in the ICU’s varies widely from 3.4% to 21% [5, 6]. The care of critically ill pregnant women requires knowledge not only of the primary disease process and its treatment in the non-pregnant women, but also a thorough understanding of the changes that the maternal peripartum physiology requires of such care [7].

## Methods

### Study Design and Patient Population

The study has a prospective, observational design. It was conducted in a general, community inner-city hospital located in North Brooklyn, New York. The hospital has a total of 363 beds distributed among General Internal Medicine, General Surgery, Psychiatry, Obstetrics/Gynecology, and Pediatrics wards. The Obstetrics/Gynecology service in this institution is composed by 25 beds, with two-dedicated operating rooms. The service has an annual average of 35,000 outpatient visits, with an approximate of 2,100 admissions per year. Nearly 1,700 newborns are delivered every year, mostly trough vaginal route. The MSICU in our institution is composed by 12 beds, with an annual admission average of 800 patients.

All obstetric patients admitted to the MSICU, either from the emergency department, the operating rooms, or from the service ward was enrolled in the study. The review included all females admitted to the ICU during pregnancy or within 42 days of delivery. Eligibility criteria included age of 18 years or older, and patients who meet the institutional requirements for admission to the MSICU. We excluded patients that were readmitted to the MSICU within 30 days. Computerized medical records were reviewed and clinical information was abstracted for each patient. Institutional Review Board approved the study.

## Results

During the 3-year study period, a total of 4,715 mothers delivered at our institution. 19 patients were admitted to the MSICU at a rate of 6-7 patients per year. The overall incidence of ICU admission was 4.1 per 1,000 deliveries. The median age was 29 years (18-41 years). Eleven (58%) patients were pregnant at the time of admission to ICU. The mean gestational age was 23.5 weeks (6-39 weeks). The most common comorbid conditions among these patients were pregnancy-induced hypertensive disorders, followed by asthma, seizures disorder, and diabetes mellitus. Conditions that required ICU monitoring are summarized in Table 1.The most common admission diagnosis was shock (9, 47%); 7 patients had hemorrhagic shock. All patients admitted with hemorrhagic shock received multiple blood transfusions (2-15 packed-red blood cells (PRBC’s) per patient), with a total of 48 PRBC’s administered; 2 (28%) patients required transfusion of fresh-frozen plasma to correct the coagulopathy resulting from massive transfusion syndrome. Three (16%) patients required ICU monitoring for respiratory failure, but none of them have needed mechanical ventilation.

**Table 1:** Admission Diagnosis Requiring ICU Monitoring

1) Shock (9, 47%)
hemorrhagic (7)
rupture of ectopic pregnancy (4)
after spontaneous abortion (1)
after dilation and curettage for incomplete abortion (1)
intrabdominal hematoma after cesarean section (1)
septic (1)
urinary tract infection
cardiogenic (1)
dilated cardiomyopathy
2) Pregnancy-induced hypertensive disorders (4, 21%)
eclampsia (3)
pre-eclampsia (1)
3) Respiratory failure (3, 16%)
hypoxemic (2)
hypercapneic (1)
4) Electrolyte imbalance (2, 10%)
diabetic ketoacidosis
5) Seizures disorder (1, 5%)
tonic-clonic, non status epilepticus

Eight (42%) patients required mechanical ventilation, with a total mechanical ventilation days of 32. There were no mechanical complications related to the use of ventilators. Almost all patients (7, 87%) were successfully weaned and extubated. A total of 4 (21%) patients needed vasoactive drugs, with total vasopressors days of 9. The total of ICU days utilized for those patients were 68. The mean length of stay was 3.5 days (1-16 days). The majority of patients (18, 95%) stayed in ICU for a period of 1-5 days, and 1 patient required ICU for 16 days. The longer duration of the ICU stay would reflect the severity of the patient’s clinical situation, 7 (36%) patients needed invasive procedures, either triple-lumen catheter and/or arterial line insertions. There were no mechanical or infectious complications resulting from those procedures. The total cost of the ICU care for those patients was $210,528, with a median cost of $9,288 ($3,096-$49,536).

There was one maternal death in MSICU during the study period. The patient was a 31 year-old African American female, 34-weeks pregnant, who was brought to the emergency department for altered mental status (found unconscious by her husband at home after unknown period of time), seizures, and elevated blood pressure. She was admitted with the diagnosis of eclampsia and possible posterior reversible encephalopathy syndrome (PRES). Brain imaging showed extensive cerebral edema and changes compatible with severe anoxic encephalopathy. An apnea test was performed and the patient was pronounced brain death.

## Discussion

Goals in management of critically ill obstetric patients involve intensive monitoring and physiologic support for patients with life-threatening but potentially reversible conditions. The occurrence of ICU admissions among obstetric patients is highly dependent on local and institutional factors in the management of these critically ill patients, and that could explained why the ICU admission rates vary in different reviews. Institutional capabilities and the frequency and acuity of serious obstetric complications largely determine the need for critical care [4]. Spiraling medical costs have prompted an evaluation of intensive care use. The concept of intermediate care or “stepdown” units was proposed for patients who did not require ICU monitoring, but who needed more care than could be provided on a general ward. It was also suggested as a strategy that promotes greater flexibility in patient triage, increases accessibility to limited intensive care, and provides a cost-effective alternative to ICU admission [8]. The availability of high-level obstetric units wherein intermediate care is offered to obstetric patients will probably influence the rates of ICU admissions. One of the advantages of high-level obstetric units is the concurrent availability of expert obstetric care and critical care management. Institutions that rely on those obstetric units for the majority of their complicated patients, mostly tertiary care centers, will transfer to the ICU only patients with multiple organ failure and those who require mechanical ventilatory support as well as invasive hemodynamic monitoring. Smaller hospitals may not be able to fulfill the requirements for an intermediate care unit, or they may not encounter enough critically ill women; in these situations, transfer to a Medical or Surgical ICU’s may be preferable for the optimal care of such patients [4]. In our study, obstetric admissions to ICU represented 4.1 per 1,000 deliveries and 2.37% of all ICU admissions in our institution, which is comparable with figures reported in the literature [5, 9, 10, 11, 12]. The mean length of stay in ICU of 3.5 days, which is similar to data reported in recent studies [10], suggested that most of these patients did not have major complications during their ICU admission. In our report, the rate of pregnancy-induced hypertensive disorders that required ICU admission was lower than in other studies [9, 10, 12] and, contrasting with previously reported data, obstetric hemorrhage was the most common diagnosis requiring admission to ICU [12, 13, 14]. The needs for mechanical ventilatory support among the patients in this study is comparable to other reports, where the necessity for mechanical ventilation ranged between 41-60% [13, 15], but it was significantly higher than in other studies [14]. Most of the patients were either transferred to ICU immediately after surgical procedures with an endotracheal tube, or intubated in the emergency department and admitted to the ICU. Our report showed that the total cost of ICU care for those obstetric patients was lower than reported in previous studies [15, 16]. Nevertheless, we presumed that some of the patients included in this review could have received optimal care in the PACU or in the intermediate care unit (step-down unit), thus reducing unnecessary admissions to the ICU. Literature on obstetric PACU admissions is lacking, while high-risk obstetric patients in non-primary obstetric hospitals are usually handle by the ICU. In those institutions the PACU may represent a suitable alternative to the intermediate obstetric unit, since the characteristics of the high-risk postoperative obstetric patients usually allow early recovery if optimal monitoring and medical treatment are readily applied. However, in hospitals where PACU is not available to deliver such care, an intermediate obstetric unit would be of a great value in accommodating such patients [17, 18].

Although the number of patients in this study was small, the mortality rate in our report was 5.2%, which is higher when compared with other reports [11, 15, 17]. It remained unclear if we should attribute this mortality to our statistics, given the critical condition and the poor prognosis of the patient on arrival to our institution. The main strength of this study was that the data was collected prospectively, over a long period of time. Furthermore, the ICU in our institution operated under a closed system that is staffed by board-certified critical care medicine physicians, thus increasing the homogeneity of clinical management and the controlling of unknown variables. Some of the limitations of our study were that it was conducted in a single, non-obstetric center and the sample size was small.

In summary, this study indicated that the reasons for critical care needs among obstetric patients have not changed over the past years. While the need for intensive care is commonly unforeseeable and unavoidable, obstetric hemorrhage and pregnancy-induced hypertensive disorders are often followed by severe maternal morbidity. Any obstetric unit knows that it will have to deal with predictable complications, even though they may occur at unpredictable times. Preparation for such emergencies, and organization of resources, may reduce the threat to maternal health and obviate the need for expensive and complex critical care. A multidisciplinary team involvement is essential in the management of pregnancy-induced hypertensive disorders and obstetric hemorrhage, as they are the most common causes of maternal morbidity and mortality as well as the main reasons for ICU admission. Understanding the physiological changes of pregnancy and the course of the diseases that commonly complicate pregnancy is essential to provide optimal quality of care. In non-primary obstetric hospitals, such as ours, taking advantage of the PACU as an intermediate obstetric unit should decreased the needs for ICU monitoring among some obstetric patients, reserving ICU beds for the most critically ill. The full scope of ICU resources should be immediately available to the obstetric patients when the need arises.
